# Sociocultural Considerations in Feeding Decision-Making Among Chinese Americans With Advanced Dementia

**DOI:** 10.1093/geroni/igaf122.1127

**Published:** 2025-12-31

**Authors:** Peiyuan Zhang, Xiaoyi Zeng, Jing Wang, Bei Wu, Dena Schulman-Green, Laura Hanson, Yaolin Pei

**Affiliations:** University of Maryland, Baltimore County, Baltimore, Maryland, United States; The University of Texas at Austin, Austin, Texas, United States; University of New Hampshire, Durham, New Hampshire, United States; NYU Shanghai, Shanghai, China (People’s Republic); New York University, New York, New York, United States; The University of North Carolina at Chapel Hill School of Medicine, Chapel Hill, North Carolina, United States; The University of Texas at Austin, Austin, Texas, United States

## Abstract

Feeding problems are part of the natural disease course among people with advanced dementia. Despite strong evidence that feeding tubes provide no clinical benefit, they remain widely used among Asian populations, including Chinese Americans, who have disproportionately high rates of feeding tube insertion. This qualitative study aimed to explore the sociocultural factors shaping the decision-making on feeding options among Chinese Americans with advanced dementia using healthcare providers’ perspectives to understand key influences on decision-making. Researchers conducted 13 individual interviews with healthcare providers who had experience caring for Chinese Americans with advanced dementia. The average duration was 60 minutes. Approximately 70% of participants had over 10 years of clinical experience. Interviews were conducted in the preferred language of participants (e.g., mandarin or English), recorded and transcribed. Deductive thematic analysis was conducted to identify key codes and themes and the research team met weekly to resolve any discrepancies. Four major themes emerged: (1) feelings of guilt over forgoing feeding tubes influenced by filial piety; (2) the positive cultural connotation of “dying with a full stomach” in traditional Chinese culture; (3) low uptake of advance care planning due to cultural taboo of discussing death and dying and limited awareness; and (4) family-centered decision-making often overriding patients’ preferences. Findings highlight the profound impact of cultural values on feeding decisions for Chinese Americans with advanced dementia. Efforts to promote culturally sensitive education and advance care planning may help support informed decision-making and align care with patients’ and families’ values.

